# New insight on microglia activation in neurodegenerative diseases and therapeutics

**DOI:** 10.3389/fnins.2023.1308345

**Published:** 2023-12-22

**Authors:** Yucong Xu, Wei Gao, Yingnan Sun, Minghua Wu

**Affiliations:** ^1^Hunan Cancer Hospital/The Affiliated Cancer Hospital of Xiangya School of Medicine, Central South University, Changsha, Hunan, China; ^2^The Key Laboratory of Carcinogenesis of the Chinese Ministry of Health, The Key Laboratory of Carcinogenesis and Cancer Invasion of the Chinese Ministry of Education, Cancer Research Institute, Central South University, Changsha, Hunan, China

**Keywords:** microglia, central nervous system, neurodegenerative diseases, circadian rhythms, drug targets, therapeutic approaches

## Abstract

Microglia are immune cells within the central nervous system (CNS) closely linked to brain health and neurodegenerative diseases such as Alzheimer’s disease and Parkinson’s disease. In response to changes in the surrounding environment, microglia activate and change their state and function. Several factors, example for circadian rhythm disruption and the development of neurodegenerative diseases, influence microglia activation. In this review, we explore microglia’s function and the associated neural mechanisms. We elucidate that circadian rhythms are essential factors influencing microglia activation and function. Circadian rhythm disruption affects microglia activation and, consequently, neurodegenerative diseases. In addition, we found that abnormal microglia activation is a common feature of neurodegenerative diseases and an essential factor of disease development. Here we highlight the importance of microglia activation in neurodegenerative diseases. Targeting microglia for neurodegenerative disease treatment is a promising direction. We introduce the progress of methods targeting microglia for the treatment of neurodegenerative diseases and summarize the progress of drugs developed with microglia as targets, hoping to provide new ideas for treating neurodegenerative diseases.

## 1 Introduction

Microglia are intrinsic immune cells of the central nervous system (CNS) that maintain CNS homeostasis. Microglia are derived from macrophages produced during hematopoiesis in the yolk sac and begin to invade the neuroepithelium at E8.5. In humans, microglia prerequisites invade the brain at the original base at approximately 4.5–5.5 gestational weeks (Paolicelli et al., 2022).

Microglia can be activated morphologically and functionally. Activated microglia are phagocytic and can act as macrophages-like to engulf cellular debris, damaged tissue, and apoptotic neurons. In addition, activated microglia release pro-inflammatory cytokines and proteases to mediate inflammatory responses and even cause neuronal damage. Microglia are categorized into several different states due to differences in the microenvironment or stimuli in which they are activated. The common ones are disease-associated microglia (DAM), axon-associated microglia (ATM), human Alzheimer’s disease microglia (HAM) and glioma-associated microglia (GAM).

Microglia are involved in neurodevelopment and disease development. Common neurodegenerative diseases include Alzheimer’s disease, Parkinson’s disease, amyotrophic lateral sclerosis, multiple sclerosis, and Huntington’s disease. Microglia are intimately involved in the onset and progression of neurodegenerative diseases. Changes in microglia phagocytosis, activation phenotyping, cellular regulatory factors, and alterations in coding genes that regulate their activity are reflected in the course of different CNS diseases.

This review summarizes the functions played by microglia in the CNS and the effects of circadian rhythms on microglia function and activation. And abnormal activation of microglia in the development of neurodevelopment and disease. In addition, this review summarizes the progress of research targeting microglia to treat CNS diseases.

## 2 The role of microglia in the central nervous system

### 2.1 Microglia maintain central nervous system homeostasis

**Figure 1:**
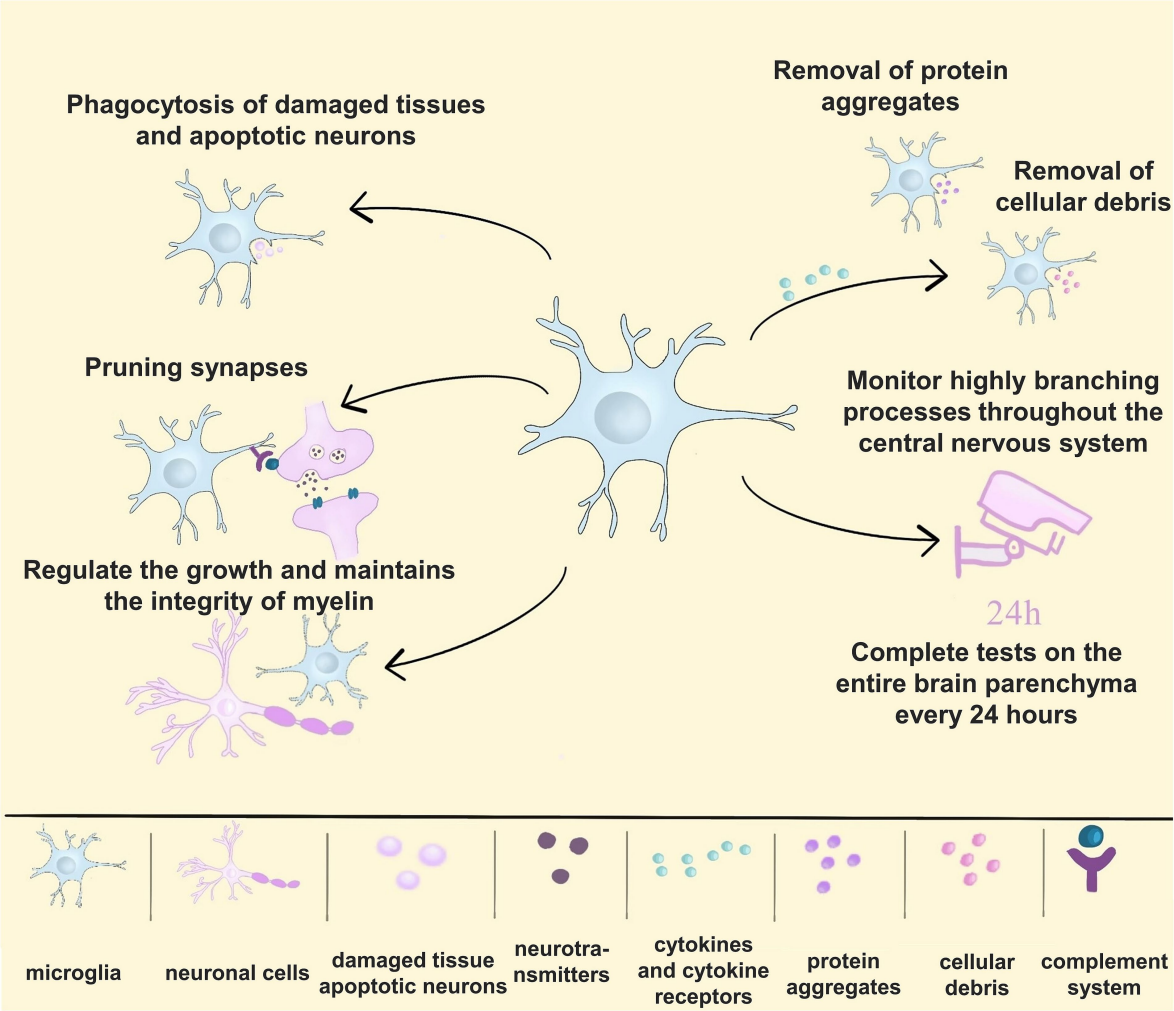
The multiple functions of microglia. (1) Microglia engulf damaged tissue and apoptotic neurons; (2) microglia can prune synapses to form proper neuronal circuits; (3) microglia regulate the growth and maintain the integrity of myelin in the central nervous system; (4) microglia secrete cytokines and cytokine receptors and remove protein aggregates and cellular debris; (5) microglia monitor highly branching processes throughout the CNS, completing a test of the entire brain parenchyma every 24 h.

Microglia are derived from the brain’s mesoderm and innate immune cells of the central nervous system involved in neurodevelopment and disease onset (Ormel et al., 2018). Genetic data indicate that microglia are key to brain health and disease (Deczkowska et al., 2018). Microglia can support neurogenesis by pruning synapses and ensuring proper neuronal circuitry in the developing brain (Schafer et al., 2012). Microglia are dispensable for myelin development, but they regulate the growth and maintain the integrity of CNS myelin (McNamara et al., 2023). Microglia can also influence the development and remodelling of the choroidal system of the central nervous system (Li et al., 2022). In the brain of a healthy adult, microglia can monitor highly branching processes throughout the central nervous system (Kyrargyri et al., 2019) and complete a test of the entire brain parenchyma every 24 h (Nimmerjahn et al., 2005). Microglia also have functions similar to macrophages to avoid brain injury and disease by engulfing damaged tissue and apoptotic neurons (Sierra et al., 2010). In addition, it can secrete cytokines and cytokine receptors and remove protein aggregates and cellular debris. Microglia play an important role in maintaining CNS homeostasis. Furthermore, microglia can resist both infectious and non-infectious damage (Prinz et al., 2019; Waltl and Kalinke, 2022). With the development of single-cell sequencing technology and genomics techniques such as genomics, transcriptomics, metabolomics, and epigenomics, researchers have entered a new stage in studying cell classification and function. In the future, historical technologies should have a bigger stage to play a crucial role in studying microglia functions and their mechanisms of action in diseases (Figure 1).

### 2.2 Activation and dynamic characterization of microglia

In previous studies, researchers have widely categorized the state of microglia into two groups: microglia under steady-state conditions and microglia that change in response to environmental signals. The former are called resting microglia, and the latter are called activated microglia. Activated microglia are widely categorized into type M1 (classically activated) and type M2 (optionally activated), with type M1 microglia being pro-inflammatory and neurotoxic and type M2 microglia being anti-inflammatory and neuroprotective (Paolicelli et al., 2022). Microglia are considered to be one of the active cells of the central nervous system. It has a range of surface receptors that detect changes in the surrounding environment. Thus, the microglia state is dynamic. Two-photon imaging shows that it extends and contracts at an average speed of about 1.5 μm/min, never truly resting (Nimmerjahn et al., 2005). In 2022, a large international team of microglia experts reached a consensus opinion against the broad and simple division of microglia into resting and activated states (Paolicelli et al., 2022).

With the widespread use of single-cell technology, research has identified microglia with several different states and phenotypes. These phenotypes can coexist and have functions that change throughout development, health, injury, and disease. In the healthy adult brain, the distribution of microglia is homogeneous, and a transcriptional continuum exists across CNS regions (Masuda et al., 2019). When a pathological environment is present, microglia switch their gene expression in response to peripheral changes, shifting to an activated state. Microglia are critical players in many neurogenic and neurodegenerative diseases. The morphological and gene expression characteristics of microglia vary in individuals with different diseases. Several microglia phenotypes have received extensive attention and research, such as DAM, ATM, HAM and GAM. DAM has received particular attention as it is one of the early identified microglia disease states. DAM is associated with phagocytosis, lysosomal, and lipid metabolism pathways and has been found in a variety of neurodegenerative disorders, such as AD and amyotrophic lateral sclerosis (ALS; You et al., 2023).

### 2.3 Neural mechanisms associated with microglia

Microglia are widely distributed in the brain and spinal cord. Microglia have a role in protecting synapses from synaptic loss and dysfunction. For example, pathologic Aβ or tau accumulating at synapses can upregulate component 1q (C1q) in microglia and promote complement activation and subsequent microglia phagocytosis at synapses. However, as the disease progresses, microglia become dysfunctional, and abnormal phagocytosis occurs, damaging neurons. This phenomenon may be specific in that microglia target specific synapses for clearance. For example, in the mouse hippocampus, a key damage sensor for microglia, expressed on myeloid cells 2 (TREM2), is involved in synaptic development. One ligand for TREM2 is phosphatidylserine (Ptdser), which is exposed outside the neuronal membrane. Thus, microglia TREM2 may sense damaged synapses in AD via PtdSer signaling (Zhong et al., 2023).

Neurodegenerative diseases vary in the extent of brain regions affected by microglia activation. Alzheimer’s disease occurs predominantly in the hippocampus, and it has been shown that activation of microglia and release of proinflammatory cytokines in patients reduces synaptic proteins in the hippocampus (Meng et al., 2023). The onset region of Parkinson’s disease is concentrated in the midbrain. In the early 1980s, McGeer observed an infiltration of activated microglia in the substantia nigra (SN) of the postmortem PD brain (McGeer et al., 1988). Results from a single-cell sequencing study of the human midbrain showed that patients with Parkinson’s disease had the most significant increase in nigral microglia. In addition, melanin idiopathic Parkinson’s disease microglia were more altered in shape, suggesting an activated state of microglia (Smajić et al., 2022). Widespread distribution of activated microglia in the brains of patients with ALS has been observed in the motor cortex, anterior frontal lobe, thalamus, and pons, among other areas. However, some regions are affected by microglia activation in various neurodegenerative diseases. Abnormal microglia activation and function alter neurogenesis in the dentate gyrus of the hippocampus, which in turn has implications for a variety of neurodegenerative diseases, including amyotrophic lateral sclerosis (ALS), Huntington’s chorea (HD), Parkinson’s disease (PD), Lewy body dementia (LBD), and frontotemporal dementia (FTD) (Terreros-Roncal et al., 2021).

There needs to be more studies on microglia’s activation and functioning regions in neurodegenerative diseases. Moreover, the areas described in the existing studies are relatively limited, and there needs to be more overall description and systematic analysis of microglia activation and functioning regions in each neurodegenerative disease. Future studies could analyze the 3D spatial location of microglia using techniques such as spatial transcriptomes. This will spatially depict information such as microglia activity trajectories and molecular characterization in the disease microenvironment, which will help search for new targets for microglia therapy.

In neurodegenerative diseases, microglia activation is mediated by multiple complex signaling pathways (Czapski and Strosznajder, 2021). In mice injected with lipopolysaccharide (LPS) to mimic an inflammatory state, the mice exhibit behavioral abnormalities of glial activation. Three days after injection, hippocampal glial cells are transiently activated, and the expression of CD68, a marker of microglia phagocytosis, is upregulated. Transient glial cell activation affects neuronal activity and behavior during the acute response phase. Ultimately, it causes systemic inflammation in mice, resulting in short-term memory deficits upon recovery (Morimoto et al., 2023). Pretreatment with low-dose LPS reduced the expression of pro-inflammatory mediators, inhibited microglia activation, and suppressed depressive-like behaviors when mice were re-stimulated with high doses of LPS (Yu et al., 2023).

Disruption of metal homeostasis alters the permeability of the blood–brain barrier, allowing for increased translocation of metals from the blood to the brain, which may exacerbate oxidative stress, and an imbalance in oxidative stress triggers a pro-inflammatory response in microglia, leading to cytokine production, which in turn affects neuronal viability and myelin production (de Oliveira et al., 2023). The cuprizone (CPZ) toxin model is commonly used to study demyelination in multiple sclerosis CPZ is a copper chelator that chelates copper cofactor-dependent mitochondrial enzymes in the respiratory chain. This causes mitochondrial dysfunction, leading to oxidative stress, which induces oligodendrocyte apoptosis. In toxin therapy, oligodendrocytes increase, microglia and astrocytes activate, and demyelination becomes evident. The activated microglia engulf the myelin sheath, causing extensive and severe axonal damage and microgliosis. Recent studies have found that the Tryptophan-Kynurenine Metabolic System is inhibited in animal models of CPZ (Polyák et al., 2023). Dysregulation of TRP-KYN metabolism is strongly associated with the neurodegenerative process, mainly through microglia activation. In treatment, researchers have observed a significant reduction of 3-hydoxykynurenine (3-HK) in brain tissue associated with microglia activity. Some studies have also reported a significant reduction in serum levels of 3-HK in patients with schizophrenia, as well as a reduction in 3-HK plasma levels in patients with major depressive disorder. These phenomena may be related to microglia activation (Cakmak et al., 2022).

Fear, stress, and depression induce the development of neurodegenerative diseases (Tanaka and Chen, 2023). These factors can induce the activation of microglia. Fear induces an elevated heart rate response, and the Neurovisceral Integration Model of Fear (NVI-f) proposes that high levels of cognitive structures (i.e., the prefrontal cortex of the brain) affect the amygdala and the hippocampus, generating a neurovisceral response by controlling the projections of cardiac behavior. Impairment of these high-level cognitive structures leads to the development of different psychiatric disorders (Battaglia et al., 2023). Neuroglial cells can be activated by stress and infections (Saijo and Glass, 2011). Especially when subjected to psychosocial stress, the activation of microglia in the amygdala and the paraventricular nucleus is consistently elevated (Calcia et al., 2016). Bright light therapy is an effective method of influencing the circadian rhythms of healthy adults, but previous studies in dementia patients have had mixed results. However, recent studies have shown that ambient bright light therapy does not improve resting-activity rhythms in patients with dementia (Kolberg et al., 2021). Diminished interleukin-6 circadian rhythms predict inhibitory symptoms and are modulated by amygdala emotional hyporesponsiveness and gene-stress interactions (Hakamata et al., 2023).

## 3 Circadian rhythms and microglia activation

### 3.1 Circadian rhythms affect microglia activation

Microglia function is regulated by circadian rhythm. Hayashi et al. found that the morphology of isolated microglia in the mouse cerebral cortex changed in response to circadian rhythms. These morphological changes were associated with protein expression cycles and the induction of purinergic receptors (Hayashi, 2013; Martínez-Tapia et al., 2020). Interestingly, Barahona et al. used inhibitors to deplete microglia and found that their neuronal network’s cortical daytime gene expression rhythm remained intact (Barahona et al., 2022). The most recent study used the CSF1R inhibitor PLX3397 to deplete 95% of microglia in the mouse brain and analyzed its effects on spontaneous behaviour in mice. They found that microglia depletion did not alter the circadian rhythm of mouse behavior (Matsui et al., 2023).

Circadian rhythms affect the activation of microglia and are closely related to the development of neuroinflammation. Microglia activation significantly alters the period and amplitude of their molecular biological clocks. The circadian rhythm in Bmal1 knockout mice is wholly lost (Brécier et al., 2023). The expression of inflammation and metabolism-related genes is significantly reduced in microglia isolated from them (Wang et al., 2020). The BV2 microglial biological clock substantially inhibits the expression of the enzyme NADPH Oxidase Isoform 2 (NOX2), which maintains a functional biological clock. NOX2 also reduces ROS and inflammatory cytokine levels and activates microglia into a pro-inflammatory state (Muthukumarasamy et al., 2023). The circadian biological clock protein Rev-erbα deficiency leads to spontaneous microglia activation, increased microglia NF-κB signaling, and neuronal injury (Griffin et al., 2019). Overactivation of microglia is thought to be an essential contributor to the development of acute neurological diseases. Lipopolysaccharide activates microglia and produces cytotoxic species such as pro-inflammatory factors, causing cellular damage. Melatonin promotes the expression of the nuclear transcription factor Nrf2 by promoting the expression of TAZ. This results in elevated Fox O1 expression, increased GSH levels, and decreased ROS levels, alleviating mouse microglia’s inflammatory response.

### 3.2 Aging affects the circadian rhythm of microglia

Aging affects the circadian rhythm of microglia and causes abnormalities in microglia function. During aging, microglia show regional heterogeneity (Grabert et al., 2016). Disturbed microglia development can cause neurodevelopmental deficits (Kwon, 2022). The number of microglia decreases markedly, and microglia depletion disrupts the circadian rhythmicity of brain tissues (Choudhury et al., 2021). Aging results in diminished circadian rhythms in microglia. One study found that the cytokines TNFα and IL-1β mRNA rhythm more strongly in the microglia of young rats. Microglia isolated from the hippocampus of aged rats express aberrant Per1 and Per2 rhythms (Fonken et al., 2016). In the aging brain, lipid droplets accumulate in senescent microglia, a phenomenon that also occurs in the presence of impaired metabolism (Marschallinger et al., 2020). Such microglia are abnormally phagocytosed and produce high levels of reactive oxygen species and secrete proinflammatory cytokines, which have been linked to the pathogenesis of neurodegenerative diseases. In addition, the connection between these senescent microglia that disrupt the blood–brain barrier (Montagne et al., 2015) and affect neurogenesis (Wu et al., 2013), which is found to be disrupted in Alzheimer’s patients after death, could be a direction for further research. Microglia Phagocytosis declines in senescent cells (Pluvinage et al., 2019) and may be defective in neurodegenerative diseases.

### 3.3 Circadian rhythm abnormalities and neurodegenerative diseases

Circadian rhythm disruption is one of the hallmarks of advanced AD, and there is also a variety of evidence suggesting that circadian rhythm disruption may be an influential factor in the pathogenesis of AD. Circadian regulation can contribute to AD progression through signaling molecules and pathways, such as the hypothalamic–pituitary–adrenal (HPA) axis- a significant mediator of stress. Among these, cortisol levels exhibit circadian fluctuations and have been observed to be elevated and dysregulated in patients with AD (Phan and Malkani, 2019). In the brain, circadian disruption leads to increased microglia activation and proinflammatory cytokine production, which results in neurogenesis deficits and reduced hippocampal synaptic proteins, ultimately impairing cognitive function (Meng et al., 2023). Insomnia-induced disruptions in circadian rhythms induce microglia activation in mice’s cerebral cortex (Phan and Malkani, 2019). Thus, circadian rhythm abnormalities contribute to microglia activation and further promote AD disease progression.

Circadian dysfunction is one of the most common symptoms of Parkinson’s, and the mechanism of circadian rhythm action in PD is closely linked to microglia. BMAL1 is an essential circadian regulator, and inactivation of brain and muscle arnt-like 1 (BMAL1) in PD model mice leads to marked motor dysfunction, loss of dopamine neurons in the dense substantia nigra, and reduction of dopamine transmitters, and allows for increased activation of microglia in the striatum In amyloid precursor protein knock-in model rats, the CLOCK/BMAL1 transcriptional negative feedback loop in microglia is impaired, and activation of the circadian clock gene Rev-erbα promotes inflammatory cytokine expression and cognitive deficits. It has also been shown that Rev-erbα deficiency exacerbates 6-OHDA-induced dopaminergic neurodegenerative disease, which is associated with the proliferation of microglia in the substantia nigra (Kou et al., 2022). Patients with PD have significantly reduced levels of circulating melatonin (MLT), and it has been demonstrated that MLT can ameliorate neuroinflammation by inhibiting signaling and transcriptional activator-associated microglial activation (Li et al., 2022).

Circadian rhythms also influence disease progression in ALS and MS. In ALS transgenic mice, circadian dysregulation accelerates disease onset and progression, demonstrating functional deficits, weight loss, and increased loss of motor neurons in the spinal cord. Expression of clock genes is altered, and the circadian rhythm-regulated gene Fus, which usually has daytime oscillatory expression, shows reduced oscillations (Zhang et al., 2018). Circadian rhythms play a crucial role in the pathology of multiple sclerosis, and groups with disrupted circadian rhythms, such as shift workers, are more likely to develop multiple sclerosis. Worsening of MS symptoms has been associated with melatonin, and although there are no abnormalities of melatonin-secreting circadian rhythms in patients with MS. However, lower levels of melatonin secretion have been associated with longer duration of MS relapses (Marin et al., 2017; Kern et al., 2019). However, the effect of circadian rhythms on disease progression by influencing microglia activation has not been reported in ALS and MS.

Huntington’s disease presents clinically with circadian rhythm disorders and sleep disturbances. In a documented study evaluating circadian rhythms in HD patients, nocturnal activity was increased in HD patients compared to controls (Hurelbrink et al., 2005). There was a delay in melatonin secretion in HD patients, which was more excellent as the disease progressed. Cortisol rhythms in patients with advanced HD showed phase advancement, increased amplitude, and increased median compared with healthy controls. Studies related to circadian rhythms have also been performed in animal models and have found a strong correlation between daytime resting activity cycle disruption and disease progression. Circadian rhythm disturbances in transgenic animal models of HD have also been correlated with gene dosage and are most prominent in animals that are homozygous for the HD-associated mutation (Nassan and Videnovic, 2022). One study found that mice treated with mesenchymal stem cells (MSC) in the R6/2 model of Huntington’s disease exhibited attenuated circadian rhythm disruption. Compared to the vector-treated group that exhibited reduced lba1 expression and altered microglia morphology, MSC treatment restored this change (Yu-Taeger et al., 2019).

Microglia have circadian rhythmicity, which is ensured by a combination of circadian core proteins and multiple circadian regulatory pathways. Dysregulation of circadian rhythms causes abnormal microglia activation and affects disease progression. Studies focus on using artificial light to regulate circadian rhythms and thereby treat disease. However, no studies have focused on regulating the abnormal activation of microglia caused by circadian rhythms to halt disease progression or reverse the disease process. Future research could delve deeper into the essential proteins or signaling pathways affecting microglia circadian rhythms and use this to find therapeutic targets for neurodegenerative diseases.

## 4 Abnormal microglia activation is a common feature of neurodegenerative diseases

Microglia play an essential role in the development of neurodegenerative diseases. Microglia play an active role in maintaining the stability of the central nervous system under normal conditions. However, when infection occurs, the part of the microglia shifts. In the onset of Parkinson’s disease and Huntington’s disease, microglia produce negative effects. Interestingly, in Alzheimer’s disease, amyotrophic lateral sclerosis, and multiple sclerosis, microglia have a dual effect. Microglia all exhibit activation abnormalities in neurodegenerative diseases, some of which are associated with gene mutations that regulate their activation or protein misfolding. Abnormal microglia activation is manifested in several ways, including abnormal numbers of microglia, altered function, and induction of neuroinflammation.

### 4.1 Microglia and Alzheimer’s disease

AD is a persistent neurological disorder that manifests in progressively more severe memory and cognitive impairment, characterized by amyloid-β (Aβ) plaque formation, neuronal cell death (Fakhoury, 2018; Shi et al., 2019; Tan et al., 2021) and microglia activation. Activated microglia not only directly mediate the central immune response but are also involved in the pathological changes of AD, including Aβ aggregation, tau protein phosphorylation, synaptic stripping, neuronal loss, and decreased memory function.

Microglia exhibit a dual role in AD. On the one hand, DAM phenotype microglia lose some of their beneficial functions and exhibit a deleterious phenotype in AD disease development. DAM is the characteristic microglial cell phenotype of AD and surrounds plaques at the site of disease (Nimmerjahn et al., 2005). Genome-wide analysis has revealed that the genes APOE4, TREM2, and CD33 have unique expression patterns in DAM (Krasemann et al., 2017; Shi et al., 2019; Zhang et al., 2023). This subtype shift is characterised by the downregulation of homeostatic genes such as Tmem119 and P2ry12 and the upregulation of DAM factors (Lpl, Ccl6, Clec7a, and Cst7). This shift implies that the cell has the required capacity to dispose of neurotoxic substances (Keren-Shaul et al., 2017; Deczkowska et al., 2018). However, microglia generated from mutations or Aβ stimulation will lose their beneficial functions, such as phagocytosis by DAMP or pathogen-associated molecular patterns (PAMPs). At the same time, these microglia exhibit deleterious phenotypes, such as the release of large amounts of neurotoxins, and progressively fail to accomplish Aβ clearance (Block and Hong, 2005). A recent study by Ennerfelt et al. described a neuroprotective microglia response driven by the signalling molecule spleen tyrosine kinase (SYK) in a model of AD disease (Ennerfelt et al., 2022; Ennerfelt and Lukens, 2023). They demonstrated that SYK is critically involved in the acquisition of DAM in neurodegenerative diseases and that the disruption of this key signalling hub in microglia disruption leads to significant defects in microglia activation, including defective Aβ phagocytosis and delayed proliferation in response to AD-related neuropathology. These studies demonstrate that enhanced SYK activation can provide a robust strategy to enhance Aβ control in mouse models of AD (Wang et al., 2022). These studies have revealed a role for SYK in the phenotypic transformation of microglia, but knowledge of key signalling molecules associated with transformation still needs to be improved. An in-depth study of the mechanisms of microglia transformation may provide new therapeutic approaches.

On the other hand, microglia exert neuroprotective effects through TREM2 receptors and CD33 receptors. The neuroprotective role of microglia is important in the study of degenerative diseases, particularly their ability to capture and phagocytose neurotoxic substances (Condello et al., 2018). According to recent studies, microglia contain and phagocytose Aβ and dead cells through the TREM2 receptor to limit the neurotoxicity produced in these pathological areas (Wang et al., 2015; Kim et al., 2017). TREM 2 is a transmembrane glycoprotein considered a protective factor in AD. It is a microglial cell Aβ receptor. Its transactivation is associated with the formation of Aβ plaques leading to AD (Colonna and Wang, 2016; Zhao et al., 2018). TREM 2 acts in combination with phospholipids and apolipoprotein E (APOE) during Aβ accumulation to regulate the proliferation, survival, and phagocytosis of associated apoptotic cells (Takahashi et al., 2005; Yeh et al., 2017). Trem2 mutations lead to impaired Aβ clearance by microglia (Zhao et al., 2018). Trem2 also negatively regulates inflammation (Thion et al., 2018; Zhou et al., 2020). A decrease in TREM 2 interferes with immune defences, leading to the accumulation of neurotoxic inflammatory factors and thus accelerating the deterioration of AD patients (Colonna and Butovsky, 2017). An increase in TREM 2 can lead to an increase in the accumulation of neurotoxic inflammatory factors. In addition, results from preclinical studies suggest that TREM 2-related microglia activation can have beneficial effects on tau hyperphosphorylation (Jiang et al., 2014; Leyns et al., 2019) and Aβ phagocytosis (Yeh et al., 2016; Parhizkar et al., 2019), and these studies demonstrate that TREM 2 may be of research value in AD treatment. On the other hand, CD33 receptors can inhibit microglia phagocytosis of Aβ (Griciuc et al., 2013), which also has a high correlation with AD risk. In addition, recent studies demonstrate that the downregulation of microglia P2Y12 receptors is closely associated with tau, which may be a sensitive indicator of neurodegenerative processes associated with Alzheimer’s disease (Maeda et al., 2021).

Excessive activation or loss of control of microglia can lead to deleterious effects of inflammatory molecules on AD-associated astrocytes (Hansen et al., 2018). Studies have shown that Aβ exposure induces the production of inflammatory cytokines that mediate the interaction between astrocytes and microglia (Elangovan and Holsinger, 2020). Excessive activation and proliferation of microglia in the vicinity of amyloid-β plaques has been observed and is associated with Aβ formation in AD. In addition, observations by Alves et al. showed that phosphorylated tau is located near microglia (Alves et al., 2019) and that NLRP 3 inflammasome activation in microglia contributes to tau pathology in AD (Lemprière, 2020). Shi et al. showed that microglia-derived cytokines in TE 4 mice could lead to A1 astrocyte activation and neuronal damage (Shi et al., 2017). Grimaldi et al. reported that activation of microglia-derived IL-1β and extracellular Aβ in the retina of AD patients triggered A1 astrocytes (Grimaldi et al., 2019). Other studies have shown that APOE is the major cholesterol transporter protein in neurons and that this protein affects APP transcription and Aβ secretion in neurons (Huang et al., 2017). In tauopathy mice, APOE 4 deficiency can be neuroprotective in tau-mediated neurodegeneration (Shi et al., 2017). In one study, it was mentioned that microglia depletion inhibited the promotion of tau pathology, whereas the increase in Aβ was closely associated with APOE 4. In addition, a review article described the importance of anti-APOE 4 immunotherapy in the therapeutic approach to AD (Williams et al., 2020). These results suggest that APOE-dependent neurodegeneration in AD can be driven by microglia. Therefore, inhibition of microglia APOE 4 may be a valuable therapeutic approach in AD.

### 4.2 Microglia and Parkinson’s disease

PD is the second most common worldwide degenerative disease of the central nervous system, occurring mostly in the elderly. It is characterized by bradykinesia, resting tremor and progressive rigidity as the main clinical features (Isooka et al., 2021). The main pathological feature of Parkinson’s disease is the death of dopaminergic neurons in the nigrostriatal system resulting in a significant decrease in dopamine released into the striatum. As the disease progresses, patients develop characteristic motor symptoms, cognitive decline, and sleep disturbances, among other neurologically related symptoms (Ho, 2019).

A critical causal factor in Parkinson’s disease is the abnormal activation of microglia due to gene mutations that regulate microglia activity. The genes encoding α-synuclein, LRRK2, PRKN, and DJ-1, which regulate microglia activity, play a role as genetic factors in the development of Parkinson’s disease. It was shown that injection of α-synuclein into the SN region of Parkinson’s disease model animals triggered microglia activation. Moreover, it was found that α-synuclein could further activate TLR, mediate nigrostriatal damage, and activate NLRP3 inflammatory vesicles and subsequent pathways that enhanced neuroinflammation and neurological damage. Studies have shown that pathogenic α-synaptic nuclear proteins promote the disease process in Parkinson’s disease (Dutta et al., 2021; Pike et al., 2021; Liu et al., 2022; Zhu et al., 2022). α-synuclein is a crucial protein involved in PD. It can act as a DAMP, leading to chronic inflammation and exacerbating neuronal dysfunction and loss by activating Toll-like receptors (TLRs). Inflammation occurs when microglia are activated and exert their phagocytosis to promote α-synuclein clearance (Basurco et al., 2023). In addition, microglia activation promotes α-synuclein aggregation and influences α-synuclein translocation in the brain.

Parkinson’s disease-associated LRRK2 mutations enhance neuroinflammatory responses, increasing dopaminergic neuron loss and motor deficits. Experiments have shown that the knockdown of the LRRK2 gene in LRRK2 (R1441G) transgenic mice inhibits the conversion of microglia to a proinflammatory phenotype (Magistrelli et al., 2022; Zhang et al., 2022). Microglia-specific regulatory chromatin regions regulate LRRK2 expression in the human frontal cortex and substantiate these results in a human-induced pluripotent stem cell-derived microglia model (Langston et al., 2022). After stimulation of LRRK2 R1441G transgenic mice using LPS, LRRK2 levels were significantly elevated in microglia. However, it was found that mRNA levels were not upregulated, which may be related to post-translational modifications. Microglia activation after LPS stimulation released more pro-inflammatory cytokines TNF-α, IL-1β, and IL-6 and anti-inflammatory cytokine IL-10 (Gillardon et al., 2012).

The PRKN gene encodes a mutated parkin protein expression inherited in an autosomal recessive manner and is usually an important genetic causative factor in early-onset familial Parkinson’s disease (Magistrelli et al., 2022). Loss of function of the parkin protein may cause abnormal mitochondrial Autophagy, and the release of inflammation-associated components in damaged mitochondria leads to activation of NLRP3 inflammatory vesicles and activation of the inflammatory response (Giorgi et al., 2021). In postmortem midbrain with the PRKN mutation, not only was dysregulation of mitochondrial DNA homeostasis confirmed but activated microglia and expression of proinflammatory cytokines were detected (Jennings et al., 2022; Wasner et al., 2022).

**Figure 2:**
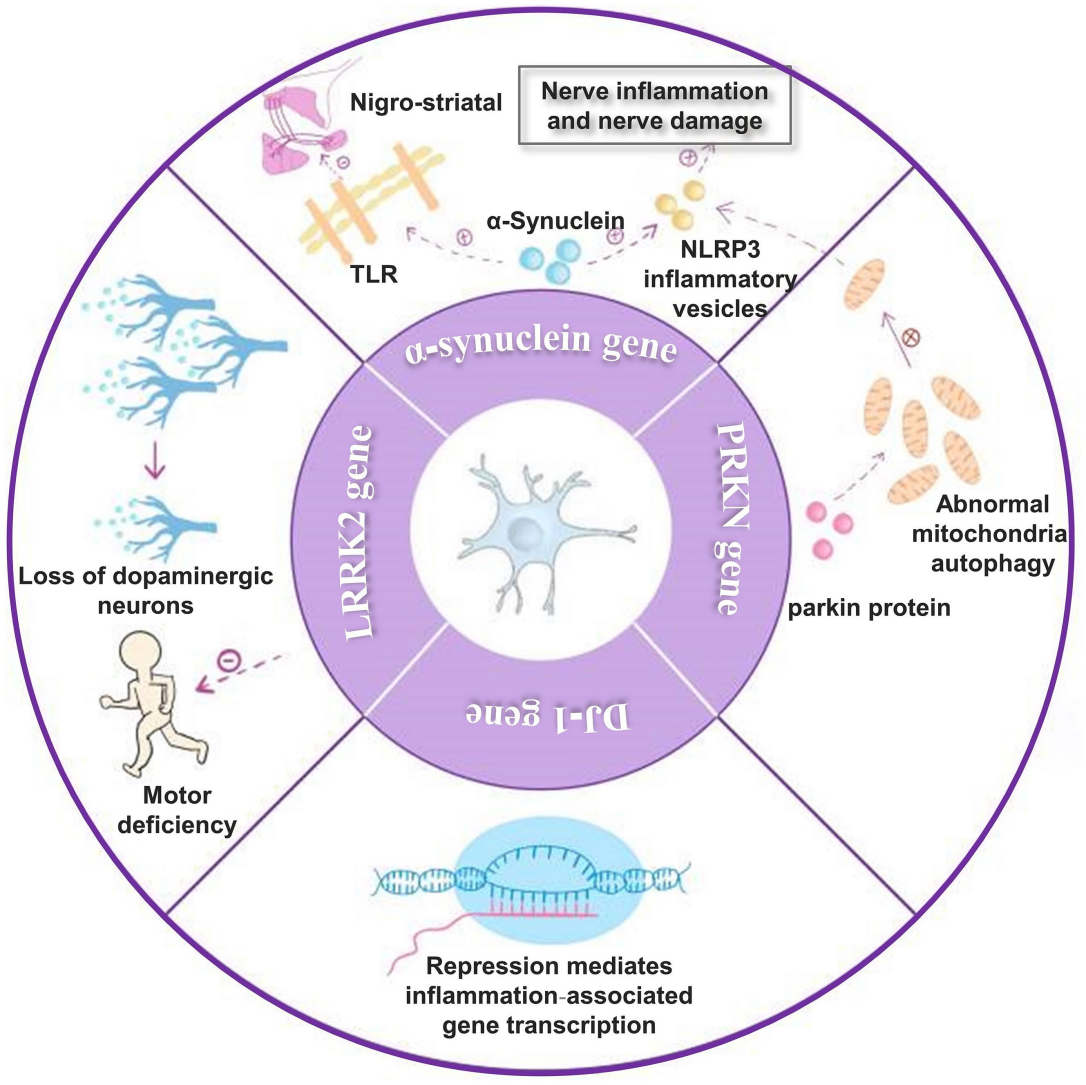
Genes that affect Parkinson’s disease. (1) α-synuclein can activate TLR and thus cause nigrostriatal damage. It also activates NLRP3 inflammatory vesicles, leading to enhanced neuroinflammation and nerve damage; (2) mutations in the LRRK2 gene enhance the neuroinflammatory response, thereby increasing dopaminergic neuron loss and motor deficits; (3) the PRKN gene encodes parkin protein and parkin protein deficiency causes abnormal mitochondrial autophagy, which leads to NLRP3 inflammatory vesicles activation and activation of inflammatory response; (4) DJ-1 gene can inhibit the transcription of genes mediating inflammation-related genes thereby decreasing microglia activation.

Mutations in the DJ-1 gene, which encodes a Parkinson’s disease-associated deglycosidase, are associated with early-onset Parkinson’s disease. DJ-1 has now been found to inhibit the transcription of genes mediating inflammation-related genes, thereby reducing microglia activation. DJ-1-deficient microglia leads to a further increase in inflammatory cytokine release. Thus, abnormal DJ-1 function leads to accelerated disease progression in Parkinson’s disease (Sliter et al., 2018; Neves et al., 2022; Pap et al., 2022; Figure 2).

Current clinical treatments cannot completely treat PD and reverse the disease process of PD. However, with the study of neuroinflammation and microglia activation, new therapeutic approaches for Parkinson’s disease, such as anti-inflammatory therapy (Ren et al., 2018; Grotemeyer et al., 2022), treatment targeting microglia polarisation (Calvello et al., 2017; Wang et al., 2019; Porro et al., 2020), and treatment targeting microglia activation (Fenster and Eisen, 2017; Liu et al., 2022; Wang et al., 2022), can be explored through anti-inflammation and immunomodulation.

### 4.3 Microglia and amyotrophic lateral sclerosis

ALS is a fatal neurodegenerative disease of the central nervous system that is difficult to recognize in its early stages (Feldman et al., 2022). ALS is sporadic, and its incidence rises with age, being highest between the ages of 60–79 years. The standardized global incidence of amyotrophic crural lateral sclerosis from the meta-analysis was only 1.68 cases per 100,000 person-years but varied by region. In populations of predominantly European descent, such as Europe and North America, the incidence rate is slightly higher than the global average, ranging from 1.71 per 100,000 to 1.89 per 100,000, and may even be higher in population-based studies (Marin et al., 2017; Kern et al., 2019).

ALS manifests as a combination of upper and lower motor neuron dysfunction affecting the medulla oblongata, cervical, thoracic, or lumbar spine. This dysfunction results in weakness of the muscles of the face, tongue, or distal upper or lower extremity muscles. This, in turn, leads to difficulty in limb movement, swallowing, speaking, and even breathing. Patients have a variety of clinical presentations. 85% of patients experience a focal episode in one body part, progressing over time to the same area on the opposite side and then to adjacent areas. There is a lack of effective treatments for ALS, and death typically occurs within 2–4 years of diagnosis of respiratory failure. Disease mortality is high, with a five-year survival rate of only 10% (Hardiman et al., 2017; Feldman et al., 2022). While existing reports have shown that multiple factors contribute to the development of ALS (Geevasinga et al., 2016), the factors that contribute to disease progression in ALS are not fully understood. Microglia play a crucial role in ALS disease pathogenesis.

Abnormal activation of microglia causes neuroinflammation to occur. Neuroinflammation and immune imbalance are essential contributors to the pathogenesis of ALS. Neuroinflammation in ALS is characterized by infiltration of lymphocytes and macrophages, activation of microglia and astrocytes, and involvement of complement. The process of immune imbalance involves infiltrating peripheral lymphocytes into the central nervous system and interacting with various immune cells, which produce different immune effects at different stages of the disease (Liu and Wang, 2017). In a normal organism, secretions from microglia distributed in the central nervous system are essential for the survival of neuronal cells, and microglia are involved in detecting and removing pathogens during the body’s immune response. Moreover, once ALS lesions occur, microglia possess the ability to damage and kill neurons. Microglia activation causes persistent neuroinflammation, which leads to the development of neurodegenerative lesions (Hickman et al., 2018). Neuronal abnormalities in the pathogenesis of ALS are associated with a decrease in microglia trophic factor secretion and the release of inflammatory factors. Microglia activation can be mediated by misfolded proteins, such as superoxide dismutase 1 (SOD1), which is associated with ALS after mutation, but not associated with wild-type SOD1 (Urushitani et al., 2006). Mutant SOD1 is secreted from neurons and astrocytes via chromogranin A and B-mediated pathways (Ezzi et al., 2007) and then efficiently activates microglia via CD14/TLR2/TLR4 and other pathways, subsequently inducing motor neuron injury.

In conclusion, in ALS patients, microglia can cause neuronal cell damage due to their abnormal secretory function on the one hand. On the other hand, the continuous activation of microglia can cause persistent neuroinflammation, which may lead to neurodegenerative lesions.

### 4.4 Microglia and multiple sclerosis

Multiple sclerosis (MS) is one of the most common central neuro demyelinating diseases (Leray et al., 2016), with a higher prevalence in women than men. The pathogenesis of MS is related to an impaired immune response (Dalakas, 2006). The impaired immune response leads to inflammatory cells invading the brain and spinal cord, resulting in CNS demyelinated areas. Inflammatory and degenerative processes destroy neurons, axons, synapses, and glial cells (O’Connor et al., 2001; Rudnicka et al., 2015; Werneburg et al., 2020). And microglia play an important role in these pathological processes.

Microglia have a dual role in the pathogenesis of MS, including both positive and negative effects (Tsouki and Williams, 2021). According to the study, microglia undergo phagocytosis and removal of myelin debris in through TREM-2, CX 3CR 11 and CD 36, which produce inhibitory effects (Lampron et al., 2015; Dong et al., 2021; Yong, 2022). On the other hand, microglia can secrete many growth factors that help restore the demyelinated areas that have occurred to their original state (Yong, 2022). Microglia exert anti-inflammatory and immunomodulatory effects through cytokines (IL-4, IL-10, TGF-β, and others) (Ponomarev et al., 2007; Brendecke and Prinz, 2015). The involvement of factors secreted by microglia in the survival, proliferation, differentiation (transglutaminase, brain-derived neurotrophic factor, insulin-like growth factor-1) and myelin formation (neuro-cilia-1) of oligodendrocyte precursor cells (OPCs) can be demonstrated in animal models or tissue cultures (Miron et al., 2013; Lloyd and Miron, 2019; Sherafat et al., 2021). Researchers have found that in MS, microglia are protective against toxins that promote pathogenic processes.

In terms of adverse effects, many compounds secreted by microglia in experimental tissue culture studies showed neurotoxic effects, such as myelencephalon-specific protease (Scarisbrick et al., 2002; Takeuchi, 2010; Mishra et al., 2014). Microglia also secrete other compounds with cytotoxic effects, including pro-inflammatory cytokines (IFN-γ, TNF-α, IL-6, IL-12) (Merson et al., 2010; Jiang et al., 2014). It has been shown that microglia can destroy oligodendrocyte progenitors through TNF-α induces oligodendrocyte death and thus destroys oligodendrocyte progenitors, while IFN-γ affects neuronal destruction (van Horssen et al., 2012; Mishra et al., 2014; Moore et al., 2015). Microglia negatively affect oxidative stress by damaging mitochondria, elevating NADPH oxidase and nitric oxide synthase, mediating ferrous recycling, and downregulating antioxidant enzymes (Bagnato et al., 2011; Haider et al., 2011; Urrutia et al., 2013; Giles et al., 2018).

Thus, changes in microglia status are involved in mitochondrial dysfunction and impaired regeneration in the CNS, thereby affecting the pathogenesis of neurodegenerative diseases (Lodygin et al., 2013; Liddelow et al., 2017; Dong and Yong, 2019). Conversely, microglia cause alterations in specific cell types through secreted cytokines that ultimately lead to injury (Elfawy and Das, 2019). Studies have shown that in MS lesions, microglia contribute to the development of neurodegenerative diseases through the release of TNF-α and IL-1 β, contributing to the generation of neurotoxic C3-positive astrocytes. These astrocytes destroy oligodendrocytes and neuroglia (Elfawy and Das, 2019). Microglia also interact with T cells, causing their abnormal activation, which can have deleterious effects (Jiang et al., 2020; Absinta et al., 2021; Wang et al., 2021). Microglia also secrete chemokines, such as growth factors that negatively affect MS (Rothhammer et al., 2018).

**Figure 3:**
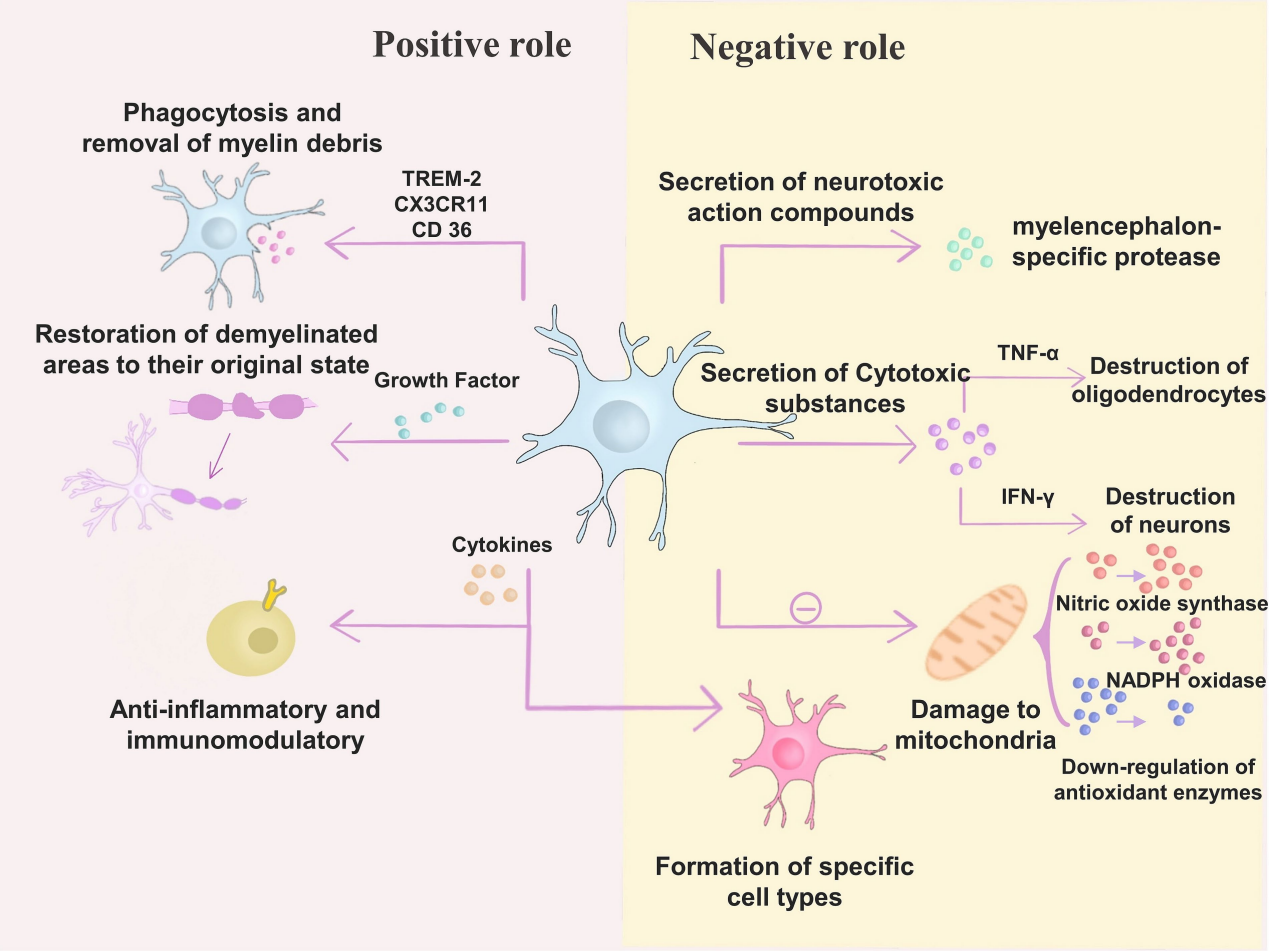
Microglia have a dual role in the pathogenesis of MS. (1) Positive role – microglia exert inhibitory effects through TREM-2, CX 3CR 11, and CD 36 for phagocytosis and removal of myelin debris; in addition, microglia can secrete many growth factors to help restore the demyelinated areas that have already occurred; microglia can also exert anti-inflammatory and Immunomodulatory effects. (2) Negative effects – microglia secrete neurotoxic compounds such as myelencephalon-specific protease; microglia secrete cytotoxic substances such as pro-inflammatory cytokines (IFN-γ, TNF-α, IL-6, IL-12). Microglia can destroy oligodendrocyte progenitors by inducing oligodendrocyte death through TNF-α, while IFN-γ has an impact on neuronal destruction; microglia can also cause changes in specific cell types through secreted cytokines, ultimately leading to injury; moreover, microglia can cause damage to mitochondria, elevate NADPH oxidase and nitric oxide synthase, as well as downregulating antioxidant enzymes.

Currently, the treatment of MS focuses on modulating the CNS’s inflammatory process. OPCs are pluripotent cells in the CNS that can differentiate into mature oligodendrocytes and act in myelin regeneration (Cignarella et al., 2020). In MS, OPCs are damaged during differentiation into mature oligodendrocytes, which leads to the onset of myelin debris deposition. In contrast, phagocytosis of microglia plays a key role in clearing the deposited myelin debris (Cignarella et al., 2020). In addition, microglia can promote myelin regeneration by secreting regenerative factors (Voet et al., 2019). This opens up opportunities for new therapeutic possibilities (Figure 3).

### 4.5 Microglia and Huntington’s disease

Huntington’s disease (HD) is an autosomal dominant neurodegenerative disorder caused by an increase in the length of CAG repeats in the Huntington gene (Ajitkumar and De Jesus, 2023). It is characterised by degeneration of striatal neurons leading to neuropsychiatric symptoms, motor deficits, and progressive cognitive impairment (Sayed et al., 2020; Stoker et al., 2022). HD is a fatal and progressive disease with an incidence of 4–10 cases per 100,000 people in Western populations and a mean age of onset of 40 years. However, it has been shown that a higher age of onset leads to a shorter survival (Langbehn, 2022). Patients exhibit uncontrolled movements of the arms, legs, and upper body (Subbarayan et al., 2022). Thirty-seven percent of patients experience phenotypic shifts and progressive loss of the ability to speak, move, think, and swallow as the disease progresses, culminating in death (Tanner et al., 2018). The disease has been shown to cause a significant increase in the number of patients who die.

In HD, abnormal activation of microglia is reflected in the number of microglia. Gene expression, microglia activation, and subsequent neuroinflammation are now considered critical features of HD and are closely associated with the development of HD (Saitgareeva et al., 2020). Expression of mHtt in microglia induces cell-autonomous increases in proinflammatory gene expression and neurotoxic effects on wild-type neurons (Lois et al., 2018). Striatal atrophy (caudate nucleus, nucleus accumbens, and pallidum) is a neuropathological feature of HD. A study noted that the number of activated microglia in the striatum and cortex directly correlated with the degree of neuronal loss. Microglia were closely associated with pyramidal neurons, suggesting that degenerating neurons induce neuroinflammatory changes (Sapp et al., 2001). Previously, Palpagama et al. described the role of activated microglial cell-derived inflammatory cytokines, ROS and quinolinic acid on neuronal death in HD (Lopez-Sanchez et al., 2020). One study found that microglia activation is an early change in HD, occurring before the onset of symptoms (Politis et al., 2015).

It has also been shown that microglia depletion in animal models ameliorates extracellular matrix changes and reduces striatal volume (Sayed et al., 2020). Microglia depletion in the R6/2 mouse model of HD prevents motor and cognitive deficits and reduces astrocyte proliferation (Petkau et al., 2019). However, selective deletion or expression of mHtt in microglia only in BACHD mice did not alter the phenotype in any way (Di Pardo et al., 2020). Treating human microglia with the longevity-associated BPIFB4 variant SV40 induced an anti-inflammatory effect of M2 polarization and prevented motor dysfunction and mHtt aggregation in R6/2 mice (Pawelec et al., 2020).

Targeting microglia as a therapeutic site for HD is a promising therapeutic direction. CX3CL1 is an essential new factor in HD pathogenesis and survival. Significantly reduced CX3CL1 gene expression in the nucleus accumbens has been observed in HD patients and mouse models. This leads to an increase in microglia-mediated synaptic pruning, resulting in dysfunctional striatal synaptic plasticity (Subbarayan et al., 2022). Thus, increasing CX3CL1 gene expression and affecting the function of microglia synapses may be a novel therapeutic strategy for HD. Galactose lectin −3 (Gal3) is a lectin that is upregulated in the plasma and brain of HD patients and mice, and their plasma Gal3 levels correlate with disease severity. Knockdown of Gal3 inhibited inflammation, reduced mHTT aggregation, improved motor dysfunction, and increased survival in HD mice. These findings suggest that Gal3 may be a novel target for the treatment of HD by inhibiting neuroinflammation, reducing mHTT aggregation, attenuating motor dysfunction, and improving survival in HD mice (Siew et al., 2019). There are no drugs against microglia for the treatment of HD, but CX3CL1 and Gal3 are extremely promising new targets.

## 5 Targeting microglia for the treatment of CNS disorders

### 5.1 Targeting microglia is an important direction in the treatment of neurodegenerative diseases

Because microglia play a phagocytic role in the central nervous system, it is thought to be a key factor in triggering neuroinflammatory responses (Kwon, 2022). Therefore, using microglia as therapeutic targets is a common approach for treating degenerative diseases of the central nervous system, such as Alzheimer’s disease, Parkinson’s disease, and amyotrophic crural lateral sclerosis (Hickman et al., 2018).

It has long been thought that the brain is an immune-privileged site where activated microglia are suppressed (Fenster and Eisen, 2017). Based on this, the first generation of therapies targeting microglia for neurological diseases was proposed, with immunosuppressive and anti-inflammatory drugs suppressing activated microglia (Kwon, 2022). Activated microglia produce factors associated with neuroinflammation, such as pro-inflammatory cytokines, chemokines and cytotoxic substances, and therefore the use of drugs targeting activated microglia may alleviate neurodegenerative diseases caused by inflammation. Non-steroidal anti-inflammatory drugs have been used in the clinical trial phase in various neurological disorders, but no clear efficacy has been reported. Clinical trials of anti-inflammatory drugs such as ceftriaxone, minocycline, erythropoietin, and valproic acid have not yielded effective results in neuroinflammatory ALS (Petrov et al., 2017). Some studies have shown that NSAIDs can prevent AD, with ibuprofen having the best effect (Vlad et al., 2008). However, ibuprofen has no role in the disease progression and treatment of AD (Jaturapatporn et al., 2012). Ren et al. performed a response meta-analysis and found that NSAIDs did not alter PD progression (Ren et al., 2018). Although no reports of effective treatment have been found, this may be related to the design of clinical treatment regimens and too rapid disease progression. From a precision medicine perspective, O’Bryant found that it was possible to select treatments for patients more sensitive to NSAID therapy and use biomarkers to observe changes in neuroinflammation as the disease progresses in anticipation of optimising treatment outcomes (O’Bryant et al., 2018). In addition, several pharmaceutical companies have turned their attention to treating neurodegenerative diseases by modulating inflammation. Canakinumab (Dhimolea, 2010) from Novartis focuses on IL-1β to treat mild cognitive impairment and Alzheimer’s by inhibiting the inflammatory response. Denali’s drug DNL788, which targets RIPK1, is currently in Phase II (NCT05237284), promising to treat ALS and MS. Minocycline is a tetracycline antibiotic. It produces anti-inflammatory effects by inhibiting inducible NO synthase, IL-1β-converting enzyme, and microglia activation (Elewa et al., 2006). A team of researchers is hoping to use small doses of interleukin-2 for immunomodulation to treat early AD in a clinical trial that has progressed to phase II (NCT05468073).

There is growing evidence that activated microglia differ according to their living microenvironment and can be classified into two polarised states: the M1 phenotype and the M2 phenotype (Michell-Robinson et al., 2015; Wang et al., 2021), which are mutually exclusive and may cause degenerative diseases of the central nervous system. The limitations of the classification of activated microglia were broken with the introduction of new microglia markers by Bennett et al. (2016) and Butovsky and Weiner (2018). Microglia fine-tune their functions according to microenvironmental, region-specific, and sex-dependent factors throughout the disease process (Chiu et al., 2013). Second-generation therapeutic approaches hope to treat CNS disorders by converting the M1 phenotype to the M2 phenotype or by improving the M2 phenotype. Dexmedetomidine is an α-2 adrenergic receptor agonist that promotes M2 polarization by inhibiting ERK1/2 signalling (Qiu et al., 2020). For example, human umbilical cord MSCs attenuate schizophrenic behaviour through IL-10 inhibition of activated microglia in mice (Yoo and Kwon, 2022). Mesenchymal stem cells (MSC) and their releasing factors convert M1-polarized microglia to an M2 phenotype via TGF-β or CX3CL1 via LPS (Noh et al., 2016). In multiple sclerosis, currently approved pharmacological approaches such as glatiramer acetate, interferon-β, fingolimod, or dimethyl fumarate may mediate microglia phenotypes from M1 to M2 polarization in part by converting neuroprotection (Giunti et al., 2014). Vitamin D can convert M1 to M2 polarised state (Boontanrart et al., 2016). The drug AL003, jointly developed by Alector and AbbVie, hopes to influence microglia activation by modulating genetic factors and cell surface receptors. Studies using MSCs in ALS and multiple sclerosis are examples of clinical trials targeting microglia phenotype conversion (Giunti et al., 2014; Oh et al., 2018).

Third-generation therapeutic approaches, based on the fact that microglia have multiple phenotypes and heterogeneity, no longer focus on altering the phenotype of microglia but more on their function (Kwon, 2022). Microglia serve as immune cells of the central nervous system, and phagocytosis of tissues is an important function of them. With age, microglia phagocytosis declines, and a tendency to synaptic pruning occurs (Deczkowska et al., 2018; Rawji et al., 2020). TREM2, a gene associated with microglia phagocytosis, is a risk factor for AD (Guerreiro et al., 2013). TREM2 deficiency prevents neuronal loss (Leyns et al., 2017). Third-generation therapeutic approaches hope to design drugs to control microglia phagocytosis by studying their phagocytic mechanisms in conjunction with impaired phagocytosis and disease progression drugs to control their phagocytic function. Gantenerumab is a human lgG1 monoclonal antibody with a high affinity for Aβ that promotes phagocytosis of aggregated Aβ by microglia. The drug has been in clinical trials since 2007 (NCT00531804). AL002 is an antibody against TREM2 developed by Alector. The drug promotes TREM2 activation, activating microglia to phagocytose Aβ, slowing AD progression. The drug was first successfully tested in clinical trials in 2018 (NCT03635047) (Wang et al., 2020). It is currently in Phase II clinical trials (NCT05744401). A team of researchers has developed the nanostructured drug pALRGT around targeting and precisely regulating the function of microglia. The drug enables microglia to phagocytose and remove myelin debris and ameliorate neuroinflammation, ultimately promoting the regeneration of myelin sheaths. The drug was explored in the neurodegenerative disease MS, and its ability to promote phagocytosis was validated (Shen et al., 2022).

Furthermore, recent studies have shown that microglia depletion and regeneration can be used to treat CNS disorders. Newly generated microglia can replace activated microglia to prevent microglia-mediated chronic inflammation and can be used to repair CNS injury (Yao et al., 2016). Methods currently used to deplete microglia *in vivo* include clodronate liposomes, genetic models, and CSF1R inhibitors. However, reducing microglia may have a dual impact on CNS degenerative diseases, traumatic diseases and defects (Shi et al., 2022).

Single-cell sequencing of microglia has shown that specific isoforms are associated with CNS disorders (Krasemann et al., 2017; Marschallinger et al., 2020). Microglia DAM isoforms are present in both mouse models of AD expressing five human familial AD gene mutations and mouse models of ALS (Keren-Shaul et al., 2017). Fifth-generation therapeutic approaches hope to investigate specific isoforms for treating CNS disorders due to the lack of subtypes for treating CNS disorders that occur due to the lack of subtypes.

**Figure 4:**
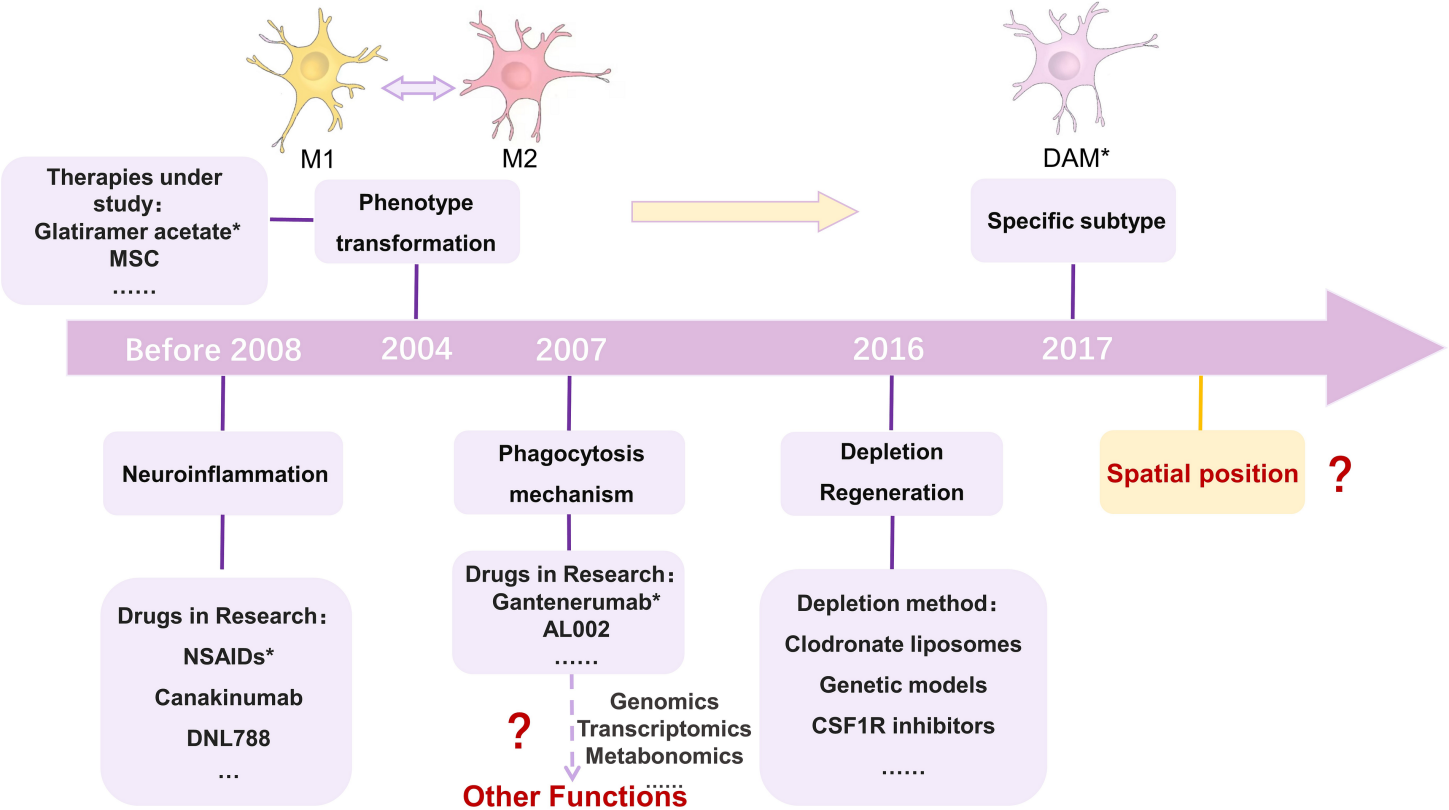
Approaches to targeting microglia for the treatment of neurodegenerative diseases. The first category inhibits activated microglia through the use of immunosuppressive or anti-inflammatory drugs; the second category of approaches looks to use drugs to alter the microglia phenotype; the third category focuses on microglia phagocytosis. With the development of histology, other functions could be focused on in the future; the fourth category of approaches focuses on the depletion and regeneration of microglia; the fifth category of approaches is an extension of the second category. Single-cell sequencing helps to observe more detailed cellular phenotypes, and the fifth category of approaches targets specific phenotypes of microglia; and the sixth category of approaches can focus on the spatial transcriptome and 3D location of microglia, which is a new direction. MSC, mesenchymal stem cells; M1, M1 microglia; M2, M2 microglia; DAM, Disease-Associated Microglia. NSAIDs were reported in 2008 to help prevent AD, but the idea for the treatment was proposed earlier than 2008 (Vlad et al., 2008). 2004 was the year in which glatiramer acetate could promote the phenotypic transformation of microglia in MS (Kim et al., 2004). Gantenerumab was first tested in a clinical trial in 2007 (NCT00531804). In 2016, *de novo* microglia could replace activated microglia to prevent inflammation and repair CNS damage (Kern et al., 2019). In 2017, DAM was discovered using single-cell sequencing (Deczkowska et al., 2018).

In neurodegenerative diseases, microglia activation is broadly similar to that of the region in which the disease occurs (McGeer et al., 1988). However, there are some regions where microglia activation is observed in multiple disease occurrences, such as the dentate gyrus of the hippocampus (Terreros-Roncal et al., 2021). In the future, the relative location of microglia in disease can be analyzed using spatial transcriptomics. Not only will it be possible to learn where microglia function, but also the mechanisms by which microglia interact with other molecules and the pathways in which they are involved. Thus, exploring the spatial location of microglia could help explore new targets for microglia in the treatment of neurodegenerative diseases (Figure 4).

### 5.2 Drugs targeting microglia

Drugs that use microglia as a target to treat neurodegenerative diseases are still in the experimental stage, and no drug has yet been applied to clinical treatment (Table 1). Several well-known pharmaceutical companies have begun to develop drugs that target microglia. The drug AL002, developed by Alector (NCT05744401), the drug DNL919, developed by Denali (NCT05450549), and the drug VGL101, developed by Vigil Neuroscience (NCT05677659), all target TREM2 for the treatment of Alzheimer’s disease. AL002 is a TREM2 agonistic antibody that activates microglia by promoting the activation of TREM2 and induces their value-addition, promoting phagocytosis of Aβ and delaying the emergence and progression of Alzheimer’s disease. Alcobra’s MG01CI (AL014), which can also be used to treat Alzheimer’s disease, targets MS4A4A as a therapeutic target (NCT02126995). Gantenerumab, developed by Roche can bind to aggregated Aβ proteins and degrade amyloid deposits by recruiting microglia and activated macrophages. Gantenerumab is an FDA breakthrough therapy for Alzheimer’s disease (NCT04592341/NCT03443973/NCT03444870/NCT05256134/NCT04339413/NCT04374253/NCT02051608). Drugs such as Canakinumab (ACZ885) (NCT04795466), AL002, and VGL101 are progressing faster, with studies moving into clinical phase II. While drugs such as AL003 (NCT03822208), DNL919 (NCT05450549), and ABBV-0805 (NCT04127695) are currently in clinical phase one.

**Table 1:** Drug for neurodegenerative diseases developed for microglia.

Drug candidate	Target	Disease	Phase	NCT
Canakinumab (ACZ885)	IL-1β	Mild cognitive impairment, Alzheimer’s	II	NCT04795466
AL002	TREM2	Alzheimer’s disease	II	NCT05744401
DNL919	TREM2	Alzheimer’s disease	I	NCT05450549
VGL101	TREM2	Alzheimer’s disease, Adult-onset leukoencephalopathy with axonal spheroids and pigmented glia	II	NCT05677659
MG01CI (AL014)	MS4A4A	Alzheimer’s disease	Preclinical	NCT02126995
AL003	SIGLEC-3/CD33	Alzheimer’s disease	Paused after Phase I	NCT03822208
JNJ-40346527	CSF1R	Alzheimer’s disease	I	NCT04121208
DNL151	LRRK2	Parkinson’s disease	III	NCT05418673
DNL201	LRRK2	Parkinson’s disease	Paused after Phase I	NCT03710707
BIIB094	LRRK2	Parkinson’s disease	I	NCT03976349
ABBV-0805	a-Synuclein	Parkinson’s disease	I	NCT04127695
ABBV-8E12	Tau	Alzheimer’s disease	Paused after Phase II	NCT02880956
Elezanumab	RGMa	Multiple Sclerosis	II	NCT03737812/NCT03737851
SAR443820 (DNL788)	RIPK1	Amyotrophic Lateral Sclerosis, Multiple Sclerosis	II	NCT05237284/NCT05630547
Gantenerumab	Amyloid-β	Alzheimer’s disease	III	NCT04592341/NCT03443973/NCT03444870/NCT05256134/NCT04339413/NCT04374253/NCT02051608

In 2017, the first small molecule LRRK2 inhibitor, DNL201, entered clinical trials (NCT03710707) and demonstrated inhibition of LRRK2 kinase activity in healthy volunteers in a Phase I trial. In 2022, Matthew et al. systematically evaluated the cascade of DNL201 in treating PD in both basic research and clinical Phase I and found that a safe dose could correct lysosomal dysfunction in PD patients (Jennings et al., 2022). However, it is unlikely that DNL201 could restore damaged or dead dopamine-producing neurons and thus reverse symptoms in PD patients. In addition, researchers at Denali evaluated the efficacy of the DNL151 small molecule drug. They found that DNL151 also inhibits LRRK2 and lasts longer in the bloodstream than DNL201, which may reduce the frequency with which patients must take the drug. As a result, clinical trials of DNL201 were stopped at this stage. In August 2023, DNL151 has completed Phase III clinical trials (NCT05418673).

In February 2017, AbbVie initiated 2 Phase II clinical research programs to evaluate the potential of an experimental anti-tau monoclonal antibody drug, ABBV-8E12, for the treatment of early AD (NCT02880956) and progressive supranuclear palsy (PSP) (NCT02985879). These two neurodegenerative diseases are very similar, with pathology characterized by increased Tau in the brain. However, in 2019, ABBV-8E12 failed miserably in Phase II of PSP. Similarly, Biogen announced that its anti-Tau antibody, Gosuranemab, failed to meet its primary endpoint in TANGO, a Phase II clinical study in AD, and that there was no therapeutic benefit of Gosuranemab compared to placebo. Gosuranemab clinical development was then terminated. The lack of success in developing these drugs illustrates the dilemma of developing drugs targeting Tau, and the Tau protein hypothesis needs to be supported by more research results.

Clinical trials require that patients are fully informed of all information relevant to them, that consent is obtained, and that participation is voluntary. If healthy volunteers are needed during the trial phase, they are informed of all potential risks and the program, and voluntary participation is ensured. Data obtained during the trial, observable phenomena, and the effects and side effects of the drug should be recorded objectively, and the trial should be terminated promptly if it is found to be harmful to the participants. When animals are used as test subjects, animal welfare should be protected following relevant regulations. Drug developers should not violate the welfare of animals or intentionally engage in behaviors that are harmful to clinical trial participants due to the need for profit.

Currently, drugs targeting microglia for treating neurodegenerative diseases fall into five main categories. The first category inhibits activated microglia through immunosuppressive or anti-inflammatory drugs, thereby reducing the production of pro-inflammatory factors, chemokines, and cytotoxic substances. This approach was proposed before researchers fully understood the brain’s immune makeup, but it is still valid. The second class of drugs is based on microglia having pro-inflammatory and anti-inflammatory phenotypes upon activation. Targeting them from a pro-inflammatory to an anti-inflammatory phenotype with drugs can alleviate or treat the disease. Although the dichotomous categorization of microglia is no longer recommended, targeting microglia phenotypic transformation to treat disease could be helpful. There are also current clinical trial cases targeting this form of treatment. The third class of drugs focuses more on the phagocytosis of microglia than on their phenotype. It is hoped that drugs can be designed to control their phagocytosis and remove unfavorable tissue or debris from the disease promptly. The fourth class of drugs was proposed with the rapid advances in single-cell sequencing to give therapeutic approaches by targeting specific microglia subtypes associated with the disease. The fifth class of drugs focuses on the depletion and regeneration of microglia, hoping to use newborn microglia instead of activated ones for disease prevention and treatment. No drugs targeting microglia for treating CNS disorders are currently available for treating a wide range of patients, but some are in clinical trials. Unfortunately, some of these drugs have been discontinued due to unsuccessful development or the discovery of better alternatives. The root cause of such incidents is still that microglia-related hypotheses still need to be proved by sufficient evidence, or there are still unfulfilled doubts about the studies related to drug development. Microglia play a crucial role in neurodegenerative diseases, so there is great potential for drug development and therapy targeting microglia. However, this still needs to be supported by many relevant research results to be obtained by researchers in the future. Future research could focus on where microglia play a role in disease, and design targeted drugs. Researchers could also use histological approaches to focus on new classifications or functions of microglia, targeting specific functions to exploit or block them.

## 6 Discussion

This study reviews the effects of microglia activation on the neurodegenerative diseases of AD, PD, ALS, MS, and HD. We describe the function of microglia and the neural mechanisms associated with them. We elucidate that circadian rhythms are essential factors influencing microglia activation and function. Circadian rhythm disruption affects microglia activation and, thus, neurodegenerative diseases. In addition, we found that abnormal microglia activation is a common feature of neurodegenerative diseases and is an integral part of disease development. Therefore, targeting microglia to treat CNS diseases is a therapeutic option. This paper presents approaches targeting microglia for treating neurodegenerative diseases and summarizes the progress of drugs developed by targeting microglia.

Microglia are intrinsic immune cells of the CNS that maintain CNS homeostasis. Microglia are actively involved in neural development. Microglia have a variety of functions, including pruning synapses, regulating myelin growth, phagocytosis, and surveillance. These functions allow microglia to be involved in the development of neurodegenerative diseases. One study analyzed 443 brains of humans who were healthy or had AD using single-nucleus RNA-sequencing (snRNA-seq) and Assay for Transposase Accessible Chromatin with high-throughput sequencing (snATAC-seq) methods. They found 12 clusters of microglia in them. The microglia were differentiated based on characteristically expressed genes and function. These microglia were categorized as neurotransmitter receptor-associated, lipid-processing-associated, unfolded protein-associated, and glycolytic state (Sun et al., 2023). Future studies of the functions and roles of microglia in neurodegenerative disorders can be done in the context of applying genomics, transcriptomics, metabolomics, and epigenomics approaches. With the development of sequencing technology, histological analysis can identify new microglia classes, thus uncovering new functions and roles of microglia. In addition, applying histologic methods to construct disease-related microglia maps can help elucidate further the pathways and specific mechanisms by which microglia play their roles.

The regions where microglia accumulate to play their roles after activation are roughly the same as the regions where neurodegenerative diseases develop. However, there are also regions where microglia activation is observed in a wide range of disease onset, such as the hippocampal dentate gyrus. In the future, more spatial transcriptomic analyses of microglia in disease could clarify the 3D spatial location of different phenotypes of microglia activation and onset of action in disease. This will spatially map the trajectories of microglia activity and their molecular characterization in the disease microenvironment, contributing to the quest for new targets for microglia therapy.

Microglia activate into different cellular states in response to changes in the surrounding environment, resulting in different morphologies and functions. Activated microglia are phagocytic and can act like macrophages to engulf cellular debris, damaged tissue, and apoptotic neurons. In addition, activated microglia release pro-inflammatory cytokines and proteases to mediate inflammatory responses and even cause neuronal damage. In the article, we discuss the new classification of microglia. With the rapid development of single-cell technology, the broad and straightforward dichotomous classification can no longer rigorously account for many problems. So, a more detailed classification method has emerged to categorize and name microglia based on their morphological and gene expression characteristics. DAM, HAM, GAM, and other microglia have been extensively studied. This shows that the classification can be more detailed with the advancement of research methods. However, the results of previous studies using the M1/M2 classification are still relevant. Future studies could look at the relationship between disease-related specific phenotypes of microglia and disease progression. For example, DAM is a microglia specific to AD, and DAM demonstrates explicitly the characteristics of AD development. In-depth study of DAM and search for therapeutic breakthroughs in specific cell types.

Circadian rhythms are one of the essential factors affecting microglia activation. Circadian rhythms regulate the morphological changes and functions of microglia. Circadian rhythms affect microglia activation and further influence the progression of neurodegenerative diseases. In the article, the progress related to AD, PD, and HD is described, and circadian rhythm abnormalities are not only an influential factor in the development of these diseases but also manifested in the aftermath of the disease, such as sleep abnormalities or disrupted circadian rhythms. Therefore, subsequent studies could focus on the molecules or signaling pathways associated with circadian rhythm abnormalities in microglia in diseases. Altering the patient’s state by modulating the expression of molecules or mediating the relevant pathways. One study has reported the circadian transcriptome of 64 tissues, including 22 brain regions in the primate baboon. The same gene is expressed in different locations with different circadian rhythms (Mure et al., 2018). When studying specific diseases, one should focus on the regions involved in developing the disease. In mediating the critical role of circadian rhythms in neurodegenerative diseases, treatments that correct the patient’s erroneous circadian rhythms, such as the administration of artificial light or the consumption of melatonin to adjust sleep, may be considered.

Abnormal microglia activation is a common feature of neurodegenerative diseases, and microglia play a positive role in maintaining CNS stability in normal conditions. However, when disease occurs, microglia hurt some diseases. In other diseases, however, microglia coexist in various states and exhibit both negative and positive effects. Microglia play a crucial role in neurodegenerative diseases, and therefore, targeting microglia for neurodegenerative diseases is an excellent therapeutic direction. This article summarizes five microglia-targeted therapeutic approaches and describes the corresponding clinical trials and drug advances. Unfortunately, there are no drugs already on the market for patient treatment. However, a variety of drugs have entered clinical trials. Drugs such as DNL201 were terminated from development due to factors such as clinical trial failure or better alternatives. Therefore, increasing research on microglia-related mechanisms in neurodegenerative diseases is fundamental and essential for drug development. The multiple mechanisms of action of microglia in neurodegenerative diseases mentioned in the text could be the target of future research on targeted drugs. However, more research support is needed to realize their transition to drugs. Whether therapeutic outcomes can be achieved by altering microglia activity, function, or number also needs to be further explored in future studies. There may be differences in the effects of drugs developed against microglia for prevention before disease onset, for early disease development, and for use in advanced disease, all of which need to gain the attention of researchers.

This article provides a more comprehensive overview of advances in microglia activation and therapeutics for neurodegenerative diseases, emphasizing the effects of circadian rhythms on them. However, the effects of aging on microglia are not described in depth. Regarding neurodegenerative diseases, there are many related states of microglia. This paper describes the role of DAM phenotype in AD. However, the various disease-associated phenotypes are not comprehensively and systematically described. Existing drugs developed by targeting microglia mostly select popular proteins of diseases. However, not focused on disease treatment by altering the circadian rhythm of microglia, which is an objective new direction.

## 7 Conclusion

This article reviews the functions of microglia, the factors influencing activation, and the impact on neurodegenerative diseases. Recent research advances are summarized, and, as a result, it is concluded that microglia as a target for treating CNS disorders is of great value and rich in theoretical support and feasibility. We summarize here the existing research progress on microglia-targeted approaches and drugs for treating neurodegenerative diseases. It is hoped that it will be of reference and enlightening significance for developing future neurodegenerative drugs. Circadian rhythms can influence microglia activation and thus disease progression, but there are no relevant drugs designed. This could be a concern to think about in future studies. I hope this paper can provide new ideas for the treatment of CNS diseases.

## Author contributions

YX: Writing – original draft, Conceptualization, Resources, Data curation, Writing – review & editing. WG: Writing – review & editing, Data curation, Supervision, Conceptualization, Investigation. YS: Writing – review & editing, Funding acquisition, Supervision, Conceptualization. MW: Supervision, Conceptualization, Writing – review & editing.
